# Maize Root Lectins Mediate the Interaction with *Herbaspirillum seropedicae* via N-Acetyl Glucosamine Residues of Lipopolysaccharides

**DOI:** 10.1371/journal.pone.0077001

**Published:** 2013-10-09

**Authors:** Eduardo Balsanelli, Thalita Regina Tuleski, Valter Antonio de Baura, Marshall Geoffrey Yates, Leda Satie Chubatsu, Fabio de Oliveira Pedrosa, Emanuel Maltempi de Souza, Rose Adele Monteiro

**Affiliations:** Department of Biochemistry and Molecular Biology, Universidade Federal do Paraná, Curitiba, Paraná, Brazil; Centre National de la Recherche Scientifique, Aix-Marseille Université, France

## Abstract

*Herbaspirillum seropedicae* is a plant growth-promoting diazotrophic betaproteobacterium which associates with important crops, such as maize, wheat, rice and sugar-cane. We have previously reported that intact lipopolysaccharide (LPS) is required for *H. seropedicae* attachment and endophytic colonization of maize roots. In this study, we present evidence that the LPS biosynthesis gene *waaL* (codes for the O-antigen ligase) is induced during rhizosphere colonization by *H. seropedicae*. Furthermore a *waaL* mutant strain lacking the O-antigen portion of the LPS is severely impaired in colonization. Since N-acetyl glucosamine inhibits *H. seropedicae* attachment to maize roots, lectin-like proteins from maize roots (MRLs) were isolated and mass spectrometry (MS) analysis showed that MRL-1 and MRL-2 correspond to maize proteins with a jacalin-like lectin domain, while MRL-3 contains a B-chain lectin domain. These proteins showed agglutination activity against wild type *H. seropedicae*, but failed to agglutinate the *waaL* mutant strain. The agglutination reaction was severely diminished in the presence of N-acetyl glucosamine. Moreover addition of the MRL proteins as competitors in *H. seropedicae* attachment assays decreased 80-fold the adhesion of the wild type to maize roots. The results suggest that N-acetyl glucosamine residues of the LPS O-antigen bind to maize root lectins, an essential step for efficient bacterial attachment and colonization.

## Introduction

*H. seropedicae* is a plant growth-promoting diazotrophic betaproteobacterium found in association with maize, sugarcane, rice, sorghum and wheat [1]–[5]. The effective stimulus of plant growth depends on an efficient colonization. However, the mechanisms of establishment of interaction between associative endophytes and plants are not fully understood. Molecules of the bacterial envelope such as flagella proteins, exopolysaccharides, adhesins and lipopolysaccharides are the first structures that contact the host, and may be required for bacterial attachment on root surfaces. In particular, several roles have been demonstrated for the LPS in plant-microbe interactions, including communication, protective barrier against plant antimicrobials and modulation of plant defense responses [6]–[9]. The lipopolysaccharides (LPS) are highly complex macromolecules found exclusively as a monolayer on the outer membrane of gram-negative bacteria [10]. These glycoconjugates are composed of three regions: the lipid A that anchors the molecule to the outer cell membrane, the core oligosaccharide and the O-antigen [11], [12]. The biogenesis of O-antigen can be divided in four stages: 1) initiation, 2) elongation/translocation/polymerization of O-antigen repeating subunits; 3) ligation of O-antigen repeating subunits to the lipid A-core, and 4) recycling of the Und-PP polyisoprenoid carrier [13]. The attachment of the O-antigen to the core is catalyzed by a membrane-bound enzyme called WaaL which is a key step for LPS biosynthesis. WaaL catalyzes the glycosidic bonding of a sugar at the proximal end of the undecaprenyl-diphosphate (Und-PP) O-antigen with a terminal sugar of the lipid A-core oligosaccharide [14]. The *H. seropedicae* WaaL protein (402 amino acids) contains 10 predicted transmembrane domains and the highly conserved Arg-215 that in *E.coli* is important for O-antigen ligation [14].

Studies with associative bacteria showed that modification of LPS reduces plant colonization and competitiveness [15]–[18], [9]. In a previous work we constructed *H. seropedicae* mutant strains in the genes *rfbB* and *rfbC*, which are involved in dTDP-rhamnose biosynthesis, the major LPS monosaccharide [19]. Mutation in the *rfb* genes drastically altered the composition of *H. seropedicae* LPS, causing the modification of the whole molecule [20]. Such rearrangements led to diminished attachment of the bacteria to maize roots, suggesting that interaction between LPS and plant receptors was required for adhesion onto roots. Mutations in lipid A biosynthesis genes are often lethal for gram-negative bacteria [21], while mutations in genes responsible for biosynthesis of monosaccharides can produce pleiotropic effects, such as decreased growth rate and altered exopolysaccharide production [9], [19]. In the present work, we showed activation of the expression of LPS biosynthesis gene *waaL* during colonization of maize root by *H. seropedicae*; we constructed a *waaL* (codes for the O-antigen ligase) mutant strain defective in the synthesis of the O-antigen portion of LPS, and showed that it is impaired in epiphytic and endophytic colonization. We have also isolated three N-acetyl glucosamine binding lectin-like proteins from maize roots (MRLs) and demonstrated their importance in LPS-dependent attachment of *H. seropedicae* to maize and wheat roots.

## Materials and Methods

### Bacterial strains and growth conditions

The bacterial strains and their relevant characteristics are listed in Table 1. *H. seropedicae* strains were grown at 30°C and 120 rpm in NFbHPN medium [23]. *E. coli* strains were grown at 37°C in LB medium [27]. Antibiotics were added at the following concentrations when required: ampicillin (Ap) 100 µg.mL^−1^ (*E. coli*); kanamycin (Km) 50 (*E. coli*) or 500 µg.mL^−1^ (*H. seropedicae*); chloramphenicol (Cm) 300 µg.mL^−1^ (*H. seropedicae*); tetracycline (Tc) 10 µg.mL^−1^ (*E. coli* and *H. seropedicae*); streptomycin (Sm) 80 µg.mL^−1^ (*E. coli* and *H. seropedicae*).

**Table 1:** Bacterial strains and plasmids used in this study.

Strains	Relevant characteristics a	Reference
*E. coli* Top 10	F^-^ *mcrA Δ(mcrr-hsd*RMS*-mcr*BC*) φ*80*lacZΔ*M15 *ΔlacX*74 *deo*R* rec*A1 *end*A1 *ara Δ*139 *Δ(ara,leu)* 7697 *gal*U *gal*K *λ* ^−^ *rps*L *nup*G *λ* ^−^	Invitrogen
*E. coli* S17.1	*recA*, *hsdR* RP4-2-*Tc::Mu-Km::Tn7*	[22]
*H. seropedicae* SmR1	Spontaneous Sm^r^ derived from type strain Z78	[23]
*H. seropedicae* LPSEB	*waaL*::Km, Sm^r^, Km^r^	This work
*H. seropedicae* pLIG	SmR1 containing pLig, Sm^r^, Tc^r^	This work
*Azospirillum brasilense* FP2	*N*-nitrosoguanidine derived from type strain Sp7	[24]
Plasmids and vectors		
pTZHSwaaL	pTZ57 containing *H. seropedicae* SmR1 *waaL* gene, Ap^r^	This work
pTZHSwaaLKM	pTZHSwaaL with *waaL* gene disrupted by Tn5 Km cassette, Ap^r^, Km^r^	This work
pSUPHSwaaLKM	*waaL::Km* Ap^r^; Km^r^; Cm^r^;	This work
pPWplig	pPW452 containing the promoter region of *waaL* upstream from *lacZ*, Tc^r^	This work
pUC4-KIXX	Ap^r^; Km^r^; cassete Tn5 Km	[25]
pSUP202	Ap^r^; Tc^r^; Cm^r^; *mob* site	[22]
pPW452	Tc^r^, promoterless *lacZ* cassette	[26]
pTZ57R/T	Ap^r^, TA cloning vector	Fermentas

a Ap  =  ampicillin; Km  =  kanamycin; Sm  =  streptomycin; Tc  =  tetracycline; Cm  =  chloramphenicol;

### DNA manipulations and mutagenesis

The plasmids used in this study are listed in Table 1. Plasmid and total DNA purification, agarose gel electrophoresis, restriction endonuclease digestion and cloning were performed according to standard protocols [27].

For *waaL* mutagenesis, the primers HSwaaL-F (5′-ctgatcggatccagcgagggagc-3′) and HSwaaL-R (5′-cgcgcagcatgccccacagg-3′) were used to amplify the *waaL* gene from *H. seropedicae* genomic DNA, and the amplicon was cloned in pTZ57R/T. The generated plasmid pTZHSwaaL was disrupted by the *nptI* cassette that confers resistance to kanamicine (Km), isolated from pKIXX. The disrupted gene was transferred to pSUP202 and this construction was electro-transformed into *E. coli* S17.1. Finally the S17.1 transformant was used to conjugate with *H. seropedicae* SmR1. The double-recombinant mutant strains were selected by kanamicyn resistance and chloramphenicol sensitivity. Insertion of the cassette in the mutant genome and the double-crossover event were confirmed by PCR analyses. The selected mutant was named *H. seropedicae* LPSEB (*waaL)*. For reporter-gene fusion plasmids, the primers HSplig-F (5′-atagcctgcagacgacgttg-3′) and HSplig-R (5′-ctcagaattcagggtggtgc-3′) were used to amplify the promoter region of the *waaL* gene, and the amplicon was cloned as a PstI/EcoRI fragment upstream from the promoterless *lacZ* from pPW452, yielding plasmid pLIG (*waaL::lacZ*). This construction was electro-transformed in *E. coli* S17.1, and transformants were used to transfer the plasmid to *H. seropedicae* SmR1 by conjugation. The plasmid-containing strains were selected and named *H. seropedicae* pLIG (*waaL::lacZ*).

### SDS-PAGE analysis of LPS

LPS for electrophoretic analysis was extracted using the proteinase K with SDS methodology as described by Balsanelli *et al*. [19]. Four microliters of LPS sample was loaded onto a 16% SDS-PAGE and detected using silver periodate oxidation staining [28].

### Plant assays

Seeds of *Zea mays* cv. BG-7049 were surface-sterilized with 1% sodium hypochlorite and 0.01% Tween 20 (USB, Cleveland, OH, USA) solution for 20 min, followed by gently shaking in 70% ethanol for 5 min. The seeds were then washed four times with sterile distilled water, transferred to water-agar (0.8%) plates, and incubated at 28°C for 72 h in the dark. After germination, each seedling was inoculated with 10^5^ cells (1 mL) of *H. seropedicae* strains for 30 minutes at 30°C. The inoculated seedlings were transferred to a hydroponic system, composed of 30 mL of plant medium without carbon source [29] and 10 g of polypropylene spheres in a glass tube, and grown at 28°C with a 12 h light period. The bacterial counts were performed immediately after inoculation to access attached bacteria and also 1, 4, 7 or 10 days after inoculation to access endophytic and epiphytic bacteria as described by Balsanelli *et al.* [19]. The results reported represent the average of at least three independent experiments. Competition assays were performed using as inoculant *H. seropedicae* wild type and *waaL* strains at 1∶1 proportion, with a total of 10, 10^2^, 10^3^, 10^4^, or 10^5^ bacteria per seedlings. Total bacterial counts were determined as described before, and the respective strains were identified by antibiotic resistance.

To investigate if outer membrane proteins were also involved in attachment, *H. seropedicae* cells were treated with non-lethal concentration of proteinase-K (5 µg.mL^−1^ for 20 minutes) and 10^5^ treated cells were inoculated per maize seedling. The assays with treated bacteria were performed in the presence of chloranfenicol 30 µg.mL^−1^ to avoid *de novo* synthesis of bacterial proteins. The proteinase-K treatment did not affect survival of *H. seropedicae* strains. To evaluate if plant proteins were necessary for bacterial attachment, we treated the maize roots with 5 µg.mL^−1^ proteinase-K for 20 minutes and then inoculated 10^5^ *H. seropedicae* cells.

Attachment inhibition assays were performed by addition of 0.5 mg.mL^−1^ of wheat germ agglutinin (WGA, Sigma) or purified maize root lectins (MRL) to 10^5^ *H. seropedicae* cells immediately before inoculating maize or wheat seedlings. For assays with wheat, seeds of *Triticum aestivum* cv. CD104 were surface-desinfected with 95% ethanol for 30 seconds and acidified hypochlorite (NaClO 0.5% v/v, HCl 0.5% v/v, 7 mM KH_2_PO_4_, Tween 80 0.01% v/v, pH 5.5) for 5 minutes. The seeds were then washed four times with distilled sterile water, and soaked for 4 hours in sterile water. The treatment with acidified hypochlorite was repeated; the seeds were then immersed in hydrogen peroxide (30% w/v) and washed with sterile water as before. The seeds were pre-germinated in water-agar plates (0.8%) at 28°C for 24 h in the dark, and then transferred to the hydroponic system. After two days of growth, the seedlings were inoculated as before, in the presence or absence of WGA and N-acetyl glucosamine. Attached bacterial counts were determined as described by Balsanelli *et al.* [19]. Attachment competition assays between *H. seropedicae* and *A. brasilense* were performed using as inoculant a mixture of *H. seropedicae* RAM4 and *A. brasilense* FP2 at 1∶1 proportion, with a total of 10, 10^2^, 10^3^, 10^4^, or 10^5^ bacteria per seedlings, in the presence or absence of N-acetyl glucosamine (0.5 mM). Total bacterial counts were determined as described before, and the respective strains were identified by antibiotic resistance and colony morphology.

### Agglutination assays

*H. seropedicae* wild type and *waaL* mutant strain were grown in NFbHPN medium until OD_600_  = 1.0. For quantitative assays, 200 µL of the bacterial culture were incubated with increasing concentrations (0–1 mg.mL^−1^) of WGA (Sigma) without shaking for 60 minutes at 30°C. Agglutination was calculated by dividing the OD_600_ of bacteria remaining in suspension by the OD_600_ of untreated bacteria and expressed as a percentage. Agglutination assays were also performed in the presence of 2% N-acetyl glucosamine or 2% of purified LPS. Negative controls were performed with increasing concentrations of BSA (Sigma) or 2% glucose. For qualitative assays, 20 µL of the bacterial culture were spotted on glass slides, mixed with 0.5 mg.mL^−1^ of BSA, WGA, purified maize root lectins either in the presence or absence of N-acetyl glucosamine (2%) and analyzed by light microscopy.

### Maize root lectins (MRL) isolation and identification

Seeds of *Zea mays* cv. BG-7049 were surface-sterilized and germinated as before. Ten grams of roots from approximately 500 plantlets were cut, frozen in liquid nitrogen and manually disrupted using a mortar and pestle. The proteins of the root powder were suspended in 20 mL of lysis buffer (10 mM Tris-HCl, pH 7.2, 150 mM NaCl, 0.5% Triton X-100), and the mixture was sonicated with 5 pulses of 15 seconds with 45 seconds intervals on ice for protein solubilization. Insoluble particles were removed by centrifugation (4000 g, 10 min, 4°C), and the extract was loaded onto a 1 mL N-acetyl-D-glucosamine-agarose (Sigma) column (1 mL.h^−1^) pre-equilibrated with the lysis buffer. The column was washed with 20 column volumes of the lysis buffer, and proteins were eluted (0.5 mL.min^−1^) using 5 mL of elution buffer (0.5 M N-acetyl-D-glucosamine, 10 mM Tris-HCl, pH 7.2, 150 mM NaCl, 0.5% Triton X-100). The eluted sample was collected in 200 µL fractions, which were precipitated with 10% TCA (Sigma), washed with acetone twice, suspended in sample buffer and analyzed by 12% SDS-PAGE (Laemmli system). For MALDI-TOF analysis protein bands were excised manually and in-gel digested overnight at 37°C with sequencing grade trypsin (Promega, Madison, USA) as described by Westermeier and Naven [30]. MS analyses and protein identification were performed according to Chaves *et al.* [31] using a MALDI-TOF Autoflex II mass spectrometer (Bruker Daltonics, Bremen, Germany) and the MASCOT program (Matrix Science).

### *waaL* expression analyses

The *H. seropedicae* pLIG reporter strain was grown in NFbHPN medium for 16 h in the presence of tetracycline. After adjusting the culture to OD_600_  = 1.0 in buffered saline, 10^8^ cells (1 mL) were used to inoculate maize grown in the hydroponic system described above and incubated at 28°C with a 12 h light period. After 24 h, bacterial cells were recovered from the liquid medium by centrifugation and attached cells were recovered from the root surface or polypropylene spheres by vortexing, followed by centrifugation. The β-galactosidase activity of the recovered cells was then measured [32]. Protein determination was carried out according to Bradford [33]. The values are expressed as nmol of ONP formed per minute per mg of protein. The results reported represent the average of at least three independent experiments. The control obtained using maize seedlings without bacteria had no detectable β-galactosidase activity.

## Results

### *waaL* mutagenesis

The *waaL* gene was chosen for mutagenesis since the O-antigen ligase is specific for LPS biosynthesis; therefore contrasting phenotypes between wild type and mutant strains would reflect the lack of O-antigen. The *waaL* gene mutagenesis was performed by *nptI* cassette insertion, and a double recombinant strain was selected and named *H. seropedicae* LPSEB. This strain has a maximum growth rate (µ_max_  = 0.315±0.010 h^−1^) similar to that of the wild-type (µ_max_  = 0.308±0.012 h^−1^).

### *H. seropedicae* LPSEB lacks the O-antigen portion of LPS

**Figure 1:**
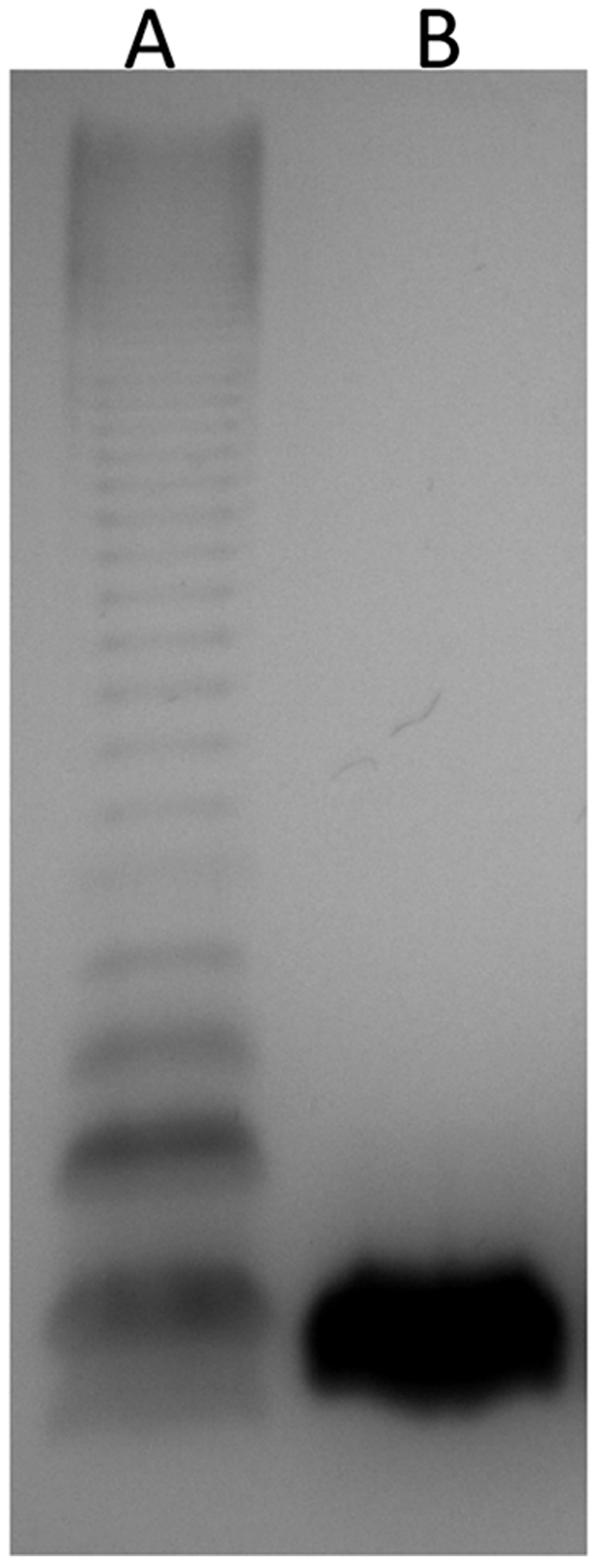
Electrophoretic pattern of LPS isolated from *H. seropedicae* SmR1 (wild type, lane A) and LPSEB (*waaL*, lane B). SDS-PAGE was performed with total LPS extracted from 10^7^ cells grown in NFbHPN medium, and LPS was visualized by periodate oxidation and silver staining.

The LPS patterns of the *H. seropedicae* strains are shown in Fig. 1. The wild type strain showed bands in a ladder-like pattern, corresponding to the lipid A-core containing different degrees of O-antigen oligomerization. On the other hand, the LPS from the mutant strain LPSEB (*waaL*) lacked the O-antigen, showed only the lipid A-core band, which had the same electrophoretic position as that of the wild type.

### *H. seropedicae* LPS O-antigen is essential for maize colonization

**Figure 2:**
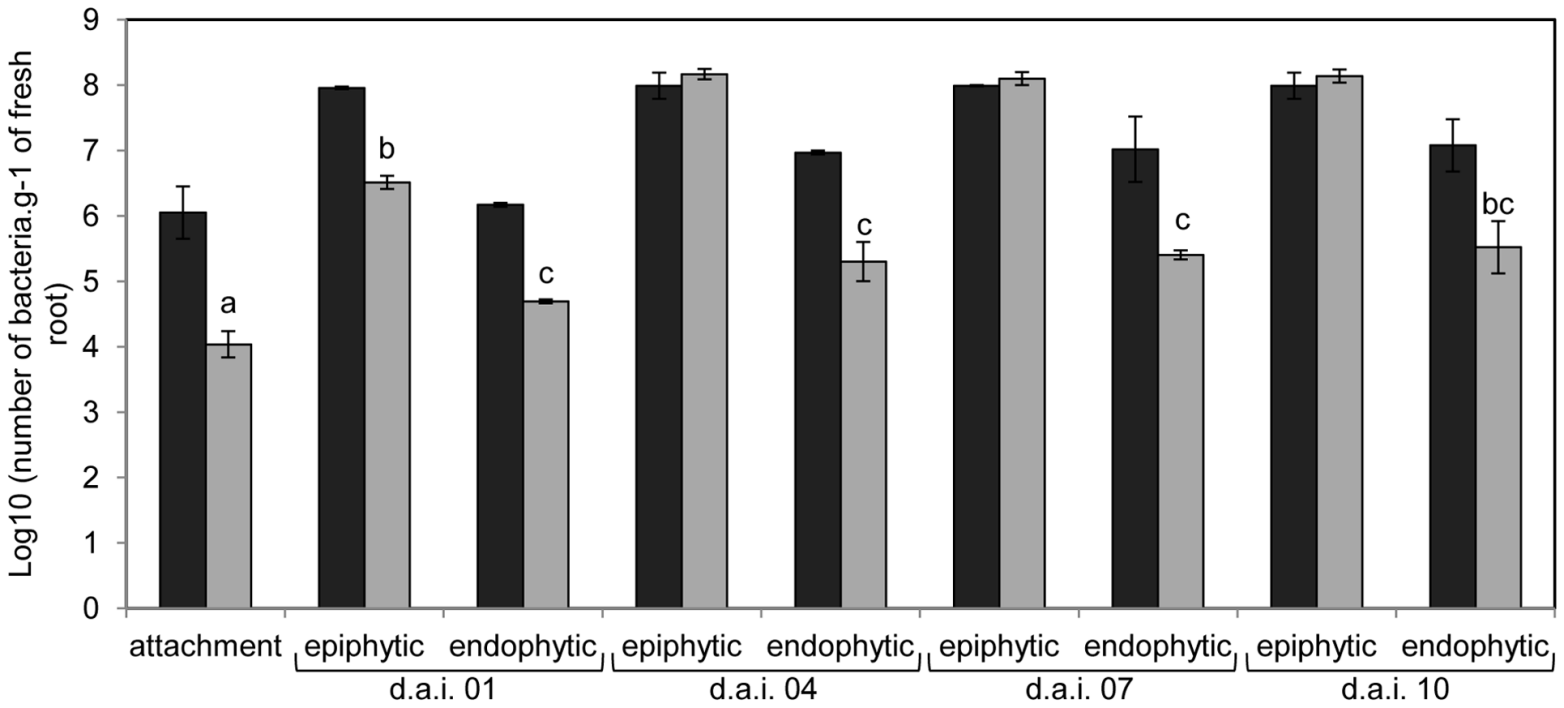
Maize root colonization by *H. seropedicae* wild-type (black bars) and *waaL* mutant (gray bars) strains. Results are shown as means of log_10_ (number of bacteria.g^−1^ of fresh root) ± standard deviation. Maize plantlets were inoculated with *H. seropedicae* strains and the number of attached bacteria determined. The numbers of epiphytic and endophytic cells were also determined 1, 4, 7 and 10 days after inoculation (d.a.i.). Different letters indicate significant differences at p<0.01 (Student *t* test).

Colonization of maize roots by *H. seropedicae* strains was followed to evaluate the LPS role in this interaction. The number of wild type *H. seropedicae* cells attached to the maize root surface was approximately 100-fold higher than that of the LPSEB mutant strain (Fig. 2). The mutant strain epiphytic population only reached that of the wild type 4 days after inoculation. The number of wild type endophytic bacteria was 100-fold higher than that of the mutant strain after 24 hours inoculation, and remained the same 10 days after inoculation (Fig. 2). These results suggest that efficient attachment and endophytic colonization of *H. seropedicae* depends on the LPS O-antigen.

**Figure 3:**
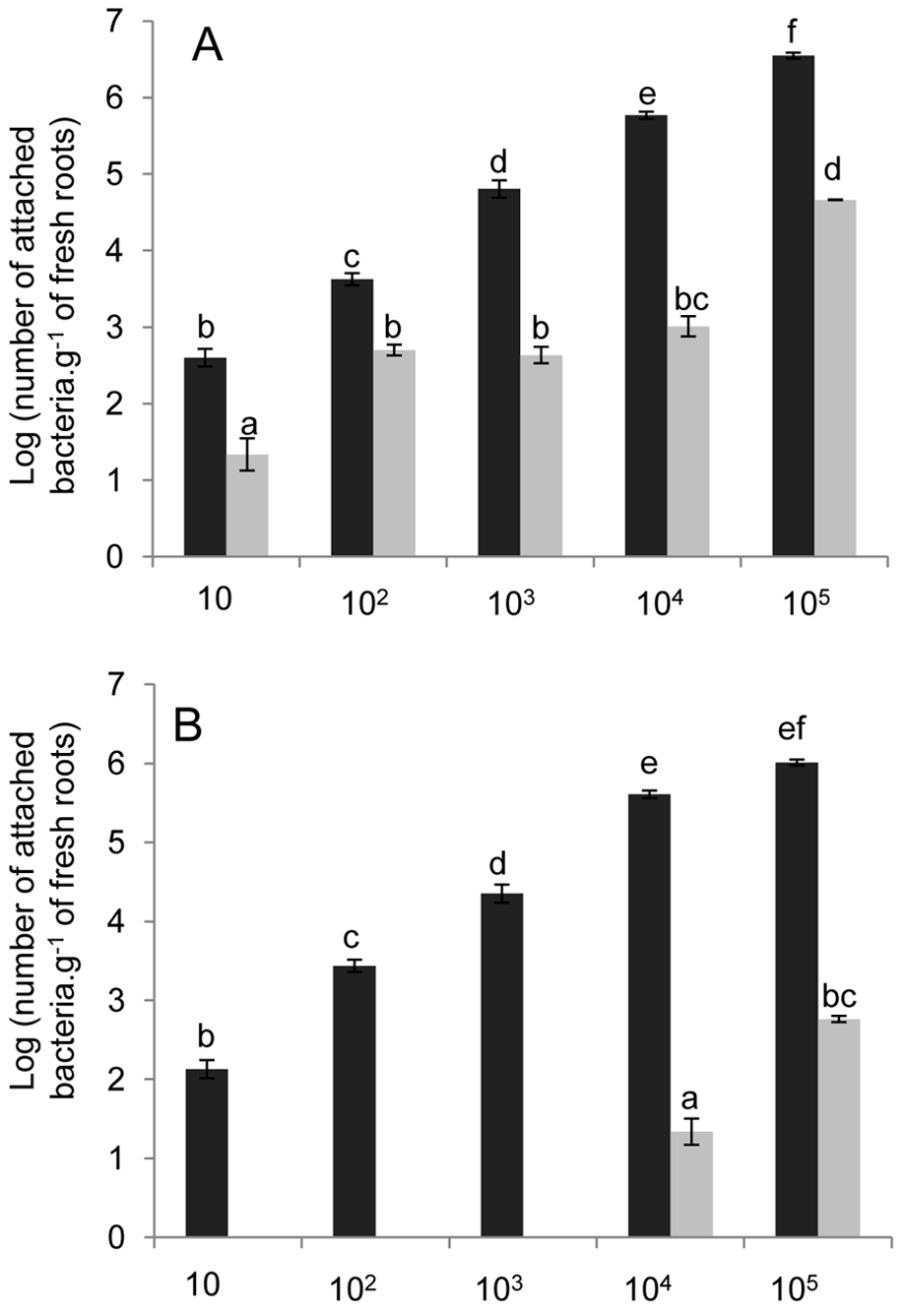
Maize root colonization by different amounts of *H. seropedicae* wild-type (black bars) and *waaL* mutant (gray bars) strains. Panel A: maize was inoculated separately with the indicated amount of each bacterial strain. Panel B: maize was inoculated with a 1∶1 mixture of both strains. The total number of bacterial cells inoculated is indicated in the x axis. Results are shown as means of log_10_ (number of bacteria.g^−1^ of fresh root) ± standard deviation. Different letters indicate significant difference at p<0.01 (Duncan multiple range test).

The use of lower numbers of bacteria for inoculation in attachment assays showed that both wild type and mutant strains had dose-dependent attachment patterns (Fig. 3A), although the numbers of the *waaL* mutant cells attached to the roots were always lower than those of the wild type. Co-inoculation assays revealed a clear predominance of the wild type strain for attachment (Fig. 3B). Inoculation of a mixture of both strains at a proportion of 1∶1 led to the recovery almost exclusively of the wild type. The mutant strain was recovered only when the inoculum used had more than 10^4^ CFU. On the other hand, wild-type colonization profile was not significantly different from that observed when it was the only strain inoculated. The fact that there was still some level of attachment of the mutant strain in most of the assays suggests the existence of an unspecific mechanism of attachment not mediated by the LPS O-antigen.

### Maize lectins mediate *H. seropedicae* attachment to root surfaces

**Figure 4:**
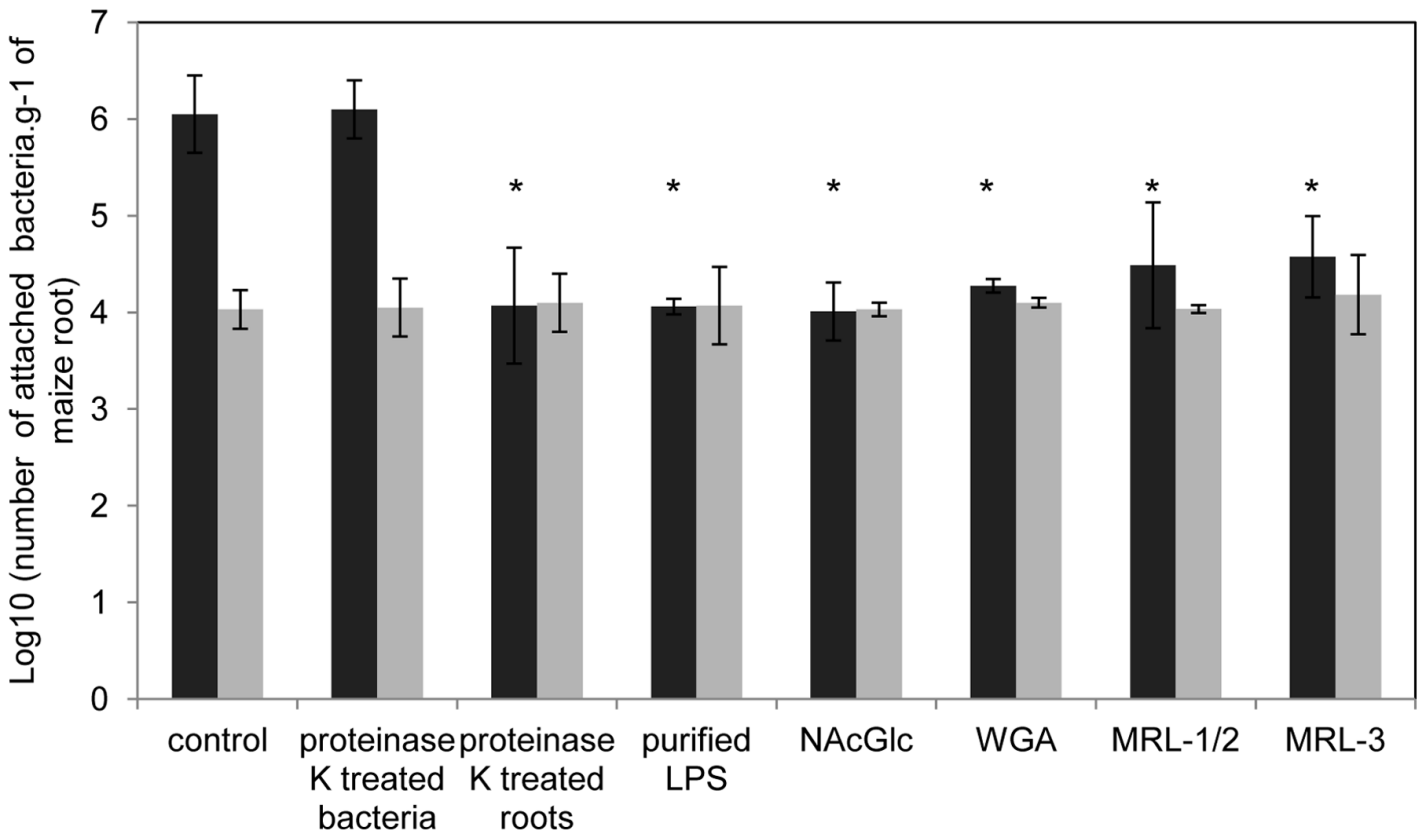
Effect of proteins on *H. seropedicae* attachment onto root surface. *H. seropedicae* wild type (black bars), *waaL* (gray bars) strains or maize roots were incubated with 5 µg.mL^−1^ proteinase-K for 20 minutes at 30°C before attachment assay where indicated. Purified LPS, N-acetyl glucosamine (NAcGlc), WGA or MRLs (0.5 mg.mL^−1^) were added as indicated. Results are shown as means of log_10_ (number of bacteria.g^−1^ of fresh root) ± standard deviation. Asterisks indicate significant difference at p<0.01 (Duncan multiple range test) of wild-type attachment between control and test conditions.

Balsanelli *et al.* [19] proposed that *H. seropedicae* LPS participates in the attachment of the bacteria by anchoring the bacterium to plant receptors by its N-acetyl glucosamine residues (28.2 mol % of O-antigen) and the results showed above reinforce the role of the O-antigen in *H. seropedicae* maize interaction. To investigate whether outer membrane proteins were also involved in attachment, the *H. seropedicae* wild type cells were treated with a non-lethal concentration of proteinase K and inoculated on maize roots. No difference in attachment was observed between treated and non-treated bacteria (Fig. 4), suggesting that bacterial external membrane proteins are not directly involved in *H. seropedicae* attachment to maize. On the other hand, when maize roots were previously treated with proteinase K and then inoculated with *H. seropedicae* the bacterial attachment decreased by 100-fold, suggesting the involvement of a plant protein in the attachment process. Moreover, similar decrease in attachment levels were also observed when the assay was performed in the presence of purified *H. seropedicae* wild type LPS or N-acetyl glucosamine, reaching attachment levels similar to that of the *waaL* mutant strain. These results strongly support the hypothesis that bacterial LPS anchors *H. seropedicae* onto plant cell surface proteins receptors.

**Figure 5:**
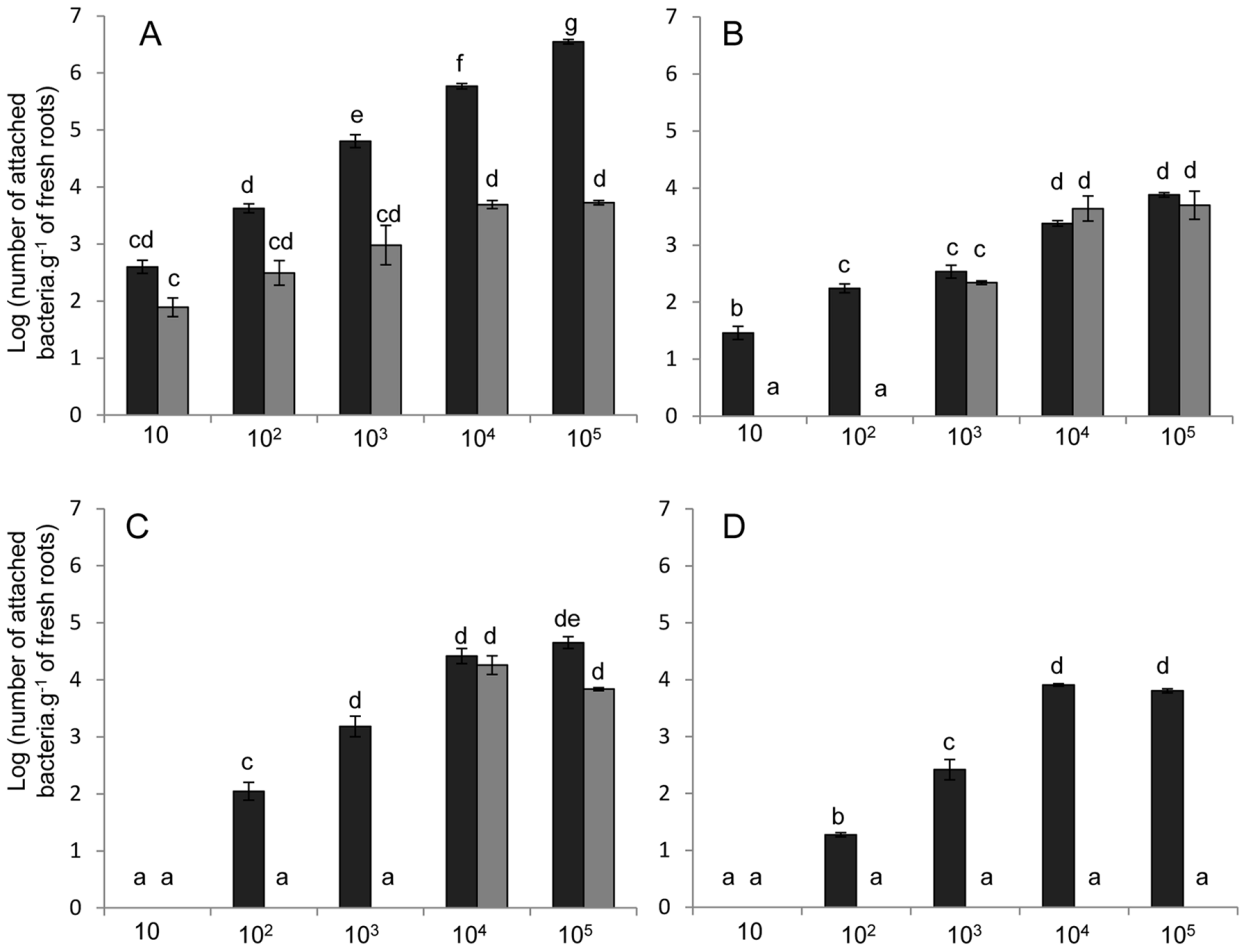
Maize root colonization by different amounts of *H. seropedicae* wild-type (black bars) and *Azospirillum brasilense* wild-type (gray bars) strains. Panel A: maize was inoculated separately with the indicated amount of each bacterial strain. Panel B: maize was inoculated with a 1∶1 mixture of both strains. Panel C: maize was inoculated separately with the indicated amount of each bacterial strain in the presence of 0.5 mM of N-acetyl glucosamine. Panel D: maize was inoculated with a 1∶1 mixture of both strains in the presence of 0.5 mM of N-acetyl glucosamine. The total number of bacterial cells inoculated is indicated in the x axis. Results are shown as means of log_10_ (number of bacteria.g^−1^ of fresh root) ± standard deviation. Different letters indicate significant difference at p<0.01 (Duncan multiple range test).

WGA is a wheat lectin, which has high affinity towards N-acetyl glucosamine, thus we tested the attachment capacity of *H. seropedicae* in its presence (Fig. 4). Addition of WGA (0.5 mg.mL^−1^) to wild type *H. seropedicae* cells suspension decreased by 100-fold the bacterial attachment to maize and wheat roots (data not showed), suggesting a conserved role of grass lectins to mediate *H. seropedicae* attachment onto root surface. *Azospirillum brasilense* also interacts with WGA, although it is not clear which outer membrane polysaccharide is involved with this binding [34]. As these organisms share a common binding mechanism, *A. brasilense* should compete with *H. seropedicae* for root attachment. Attachment assays showed that *A. brasilense* population reaches the equilibrium at 10^4^ cells per gram of root, while *H. seropedicae* showed a dose-dependent attachment pattern with maximum of 10^7^ cells per gram of root (Fig. 5A). Co-inoculation assays revealed an outstanding reduction in attachment on both strains, suggesting a competition for binding onto maize root surface (Fig. 5B). *H. seropedicae* number of attached cells was 100 to 1000-fold smaller in competition compared to isolated inoculation in all doses of inoculum. *A. brasilense*, on the other hand, was not recovered from root surface during competition when less than 10^3^ cells were used in the inoculum, but showed no difference in attachment comparing to isolated inoculation in the bigger inoculum doses. These results suggest that the bacterial species initially compete for the same attachment sites, but when there are more bacteria *A. brasilense* is able to adhere normally. The addition of GlcNAc during the attachment assays of the species (Fig. 5C) showed that *A. brasilense* attachment is blocked by this monosaccharide when less than 10^4^ cells were used in the inoculum, and again showed no difference in attachment comparing to isolated inoculation in the bigger inoculum doses. The addition of GlcNAc during the competition assays between the species (Fig. 5D) prevented completely attachment of *A. brasilense*. The attachment pattern of *H. seropedicae* in the presence of GlcNAc was similar as in the separated inoculation as during competition with *A. brasilense*, suggesting that the competition for attachment between the species is due to sharing a conserved mechanism to bind to GlcNAc-plant receptors.

WGA was also able to agglutinate *H. seropedicae* wild type cells, but not *waaL* mutant, in a concentration dependent manner, indicating that the lectin is capable of binding to the *H. seropedicae* cell surface (Fig. S1). Moreover, addition of N-acetyl glucosamine or LPS inhibited *H. seropedicae* agglutination suggesting that the binding occurs through the O-antigen region of wild type LPS. To identify receptor molecules on roots surface, total maize root proteins were extracted and lectin-like molecules were purified by affinity chromatography on an N-acetyl glucosamine-agarose column (Fig. S2). Two proteins fractions (A and B) were eluted using 0.5 M N-acetyl glucosamine. Fraction A showed two major protein bands (35 kDa and 34 kDa) which were named MRL-1 and MRL-2. Fraction B showed one band (38 kDa) indicated as MRL-3. These bands were analyzed using tryptic peptides identified by MALDI-TOF mass spectrometry (Table 2). Proteins MRL-1 and MRL-2 are similar to uncharacterized jasmonate-induced maize proteins. Both proteins have a dirigent-like domain, that is expressed in plants during disease-resistance response and lignification [35], [36], and a jacalin-like domain with six sugar binding sites which may bind mono- or oligosaccharides with high specificity [37] (Fig. S3). MRL-3 had been previously identified as uncharacterized maize protein and contains a B chain domain, a two-domain lectin with aromatic residues that can interact with the nonpolar face of saccharides and polar residues for hydrogen bond formation to the sugar hydroxyl groups [38] (Fig. S3). MRL-1 shares 74% of identity with MRL-2, but MRL-3 shares no significant similarity with MRL-1 and MRL-2. These three proteins had not been previously characterized.

**Table 2:** Identification of isolated maize root lectin-like (MRL) proteins by MALDI-TOF spectrometry.

	MRL-1	MRL-2	MRL-3
**PMF match**	B4FN23_MAIZEPutative uncharacterized protein; Jasmonate-induced protein	B4F7S2_MAIZEPutative uncharacterized protein; Jasmonate-induced protein	B4G0K5_MAIZEPutative uncharacterized protein
**Sequence coverage**	28%	18%	41%
**Identity** a	-gb|EES17612.1| hypothetical protein SORBIDRAFT_09g001880 [*Sorghum bicolor*] (63%)-gb|AAR20919.1| jasmonate-induced protein [*Triticum aestivum*] (41%)-gb|ABB51090.1| mannose-specific jacalin-related lectin [*Oryza sativa* Japonica Group] (40%)	-gb|EES17612.1|hypothetical protein SORBIDRAFT_09g001880 [*Sorghum bicolor*] (62%)-gb|AAR20919.1| jasmonate-induced protein [*Triticum aestivum*] (44%)-gb|ABB51090.1| mannose-specific jacalin-related lectin [*Oryza sativa* Japonica Group] (44%)	-gb|EER92327.1| hypothetical protein SORBIDRAFT_01g036580 [*Sorghum bicolor*] (92%)-gb|ABF95713.1| QXW lectin repeat family protein [*Oryza sativa* Japonica Group] (45%)-gb|ABW73994.1| lectin 2 [*Euonymus europaeus*] (42%)

a Protein alignment performed with NCBI PSI-Blastp (http://ncbi.nlm.gov/BLAST/).

Peptide mass fingerprint (PMF) comparisons to the database and sequence coverage data were obtained using the MASCOT program.

The MRL proteins were tested as competitors in *H. seropedicae* attachment to maize roots. The presence of MRL-1 and 2 or MRL-3 (0.5 mg.mL^−1^) decreased wild type attachment 80-fold (Fig. 4), suggesting that they constitute sites on the root surface involved in *H. seropedicae* attachment.

**Figure 6:**
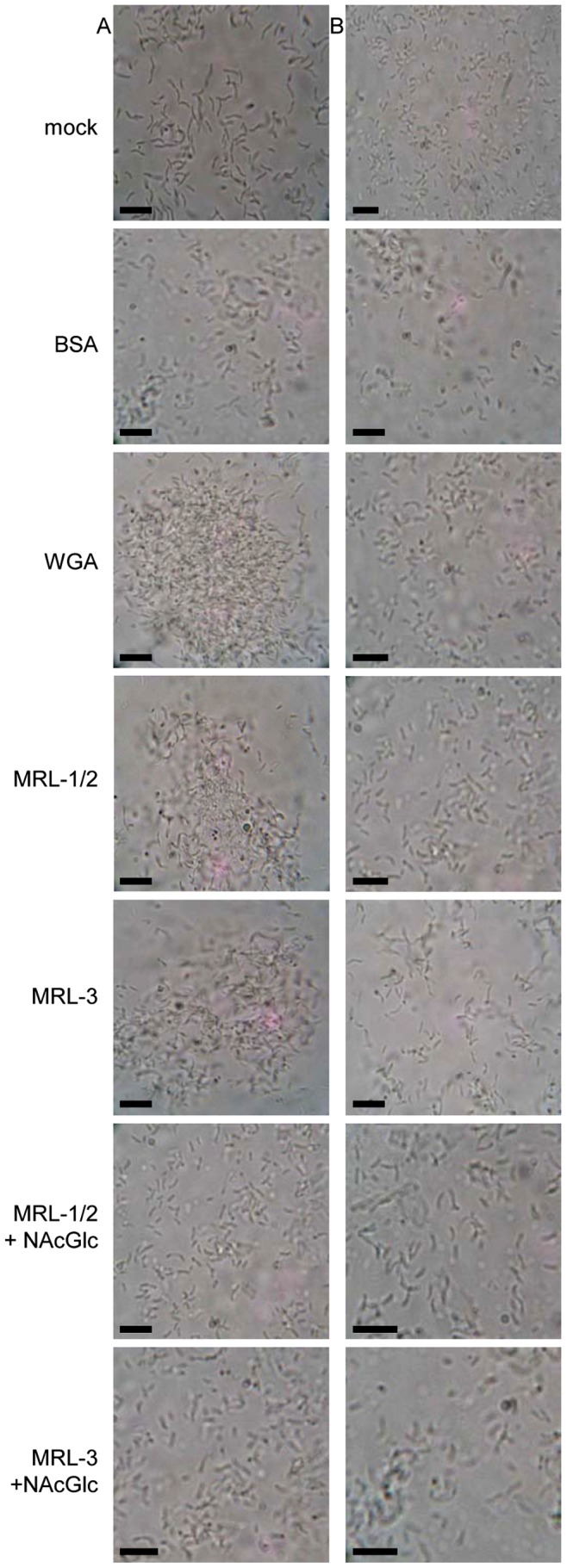
Light microscopy of *H. seropedicae* wild type (left) and *waaL* (right). Cell cultures were spotted on glass slides, mixed with bovine serum albumin (BSA), WGA, or purified maize root lectins MRLs (all 0.5 mg.mL^−1^) in the absence or presence of N-acetyl glucosamine (2%) and analyzed by light microscopy. Mock in the presence of saline buffer; bars  = 10 µm.

Fraction containing MRL-1 and MRL-2 (0.5 mg.mL^−1^), or MRL-3 (0.5 mg.mL^−1^) were also tested for agglutination activity against *H. seropedicae* (Fig. 6). Both fractions showed agglutination activity against the wild type strain, in a level similar to that observed with WGA. In both cases agglutination activity was severely reduced in the presence of N-acetyl glucosamine. In contrast, no agglutination was observed in the *waaL* mutant in the presence of any lectin.

### *H. seropedicae* attachment to maize root surface induces WaaL expression

**Figure 7:**
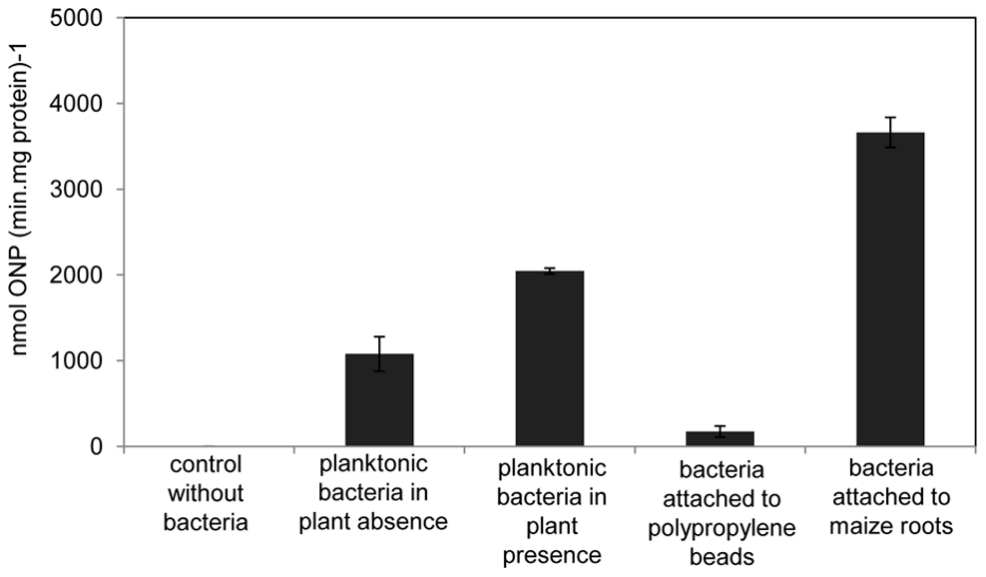
Expression of *H. seropedicae waaL* gene during interaction with maize. *H. seropedicae* carrying pLIG plasmid (*waaL::lacZ*) (10^8^ cells) were inoculated in maize plantlets in glass tubes containing 30 ml of plant medium and incubated for 24 hours at 28°C with 12 h light period. Bacterial cells from the hydroponic medium were collected by centrifugation and their β-galactosidase activity was determined. The cells attached to roots or to polypropylene beads (unspecific attachment control) were removed by vortexing, recovered by centrifugation and then the β-galactosidase activity was determined. Specific activity is expressed as nmol ONP min^−1^ (mg protein)^−1^ ± standard deviation.

In order to evaluate *waaL* expression during interaction with the host, the *H. seropedicae* strain containing a *waaL:lacZ* fusion (plasmid pLig) was inoculated into maize plantlets grown in the hydroponic system and the specific β-galactosidase activities of planktonic and root attached bacteria was determined. Compared to *H. seropedicae* cells cultivated in the absence of maize root, the expression of *waaL* was 2 folds higher in *H. seropedicae* planktonic cells, and 3.5 folds higher in maize root attached cells (Fig. 7). On the other hand, cells attached to polypropylene spheres shows background β-galactosidase. The difference in β-galactosidase activities between cells attached to maize roots and to polypropylene spheres was about 10 times higher. These results imply that induction of LPS genes expression occurs in response to specific signals from the plant. Therefore, these results indicate that plant signals induce O-antigen ligase expression, suggesting that LPS synthesis is probably induced during interaction with maize, stressing the importance of this molecule during the first steps of the host colonization.

## Discussion

Attachment of soil bacteria to plant root cells is an early step required in associative interactions. We previously reported that intact LPS is required for *H. seropedicae* attachment and endophytic colonization of maize roots, and suggested that it is necessary for the bacteria to bind to plant receptors and to resist plant chemical defenses [19]. In the present study, we show that the presence of the O-antigen portion in the LPS is essential for an efficient bacterial colonization, a role supported by the induction of LPS biosynthesis gene expression during rhizoplane colonization. The results support the hypothesis that *H. seropedicae* uses LPS to attach or adhere to plant protein receptors possibly by recognition and binding to maize root lectins.

Plant assays using a mutant in the O-antigen ligase gene (*waaL*) showed that *H. seropedicae* LPS O-antigen is necessary for an early epiphytic attachment and colonization. Moreover, impairment of the endophytic colonization caused by the lack of O-antigen was observed even ten days after inoculation. This may suggest an important role of LPS against plant basal defenses. In other rhizobacteria such as *Pseudomonas fluorescens, Azospirillum brasilense* and *R. tropici* CIAT899 alterations in the LPS structure also reduced rhizospheric and endophytic root colonization [15]-[18], [9], but the role of LPS in such interactions is not clear.

Our data suggests that bacterial surface proteins are not required for the initial attachment of *H. seropedicae* to maize roots (Fig. 4), a role that seems fulfilled by the bacterial LPS. On the other hand, the presence of maize root surface proteins enhances *H. seropedicae* attachment. Moreover, fractions containing three maize root lectin-like proteins recovered from a N-acetyl-D-glucosamine-agarose column inhibited wild type *H. seropedicae* attachment to maize roots. The WGA lectin also inhibited attachment of wild type *H. seropedicae* to maize and wheat plants, but not that of LPS mutant. We propose a model in which the N-acetyl glucosamine residues of the LPS O-antigen binds to the maize root lectins (MRL1, 2 and 3). Lectins exposed on root surfaces thus interact with bacteria in the rhizoplane due to their ability to distinguish between sugar moieties of bacterial polysaccharides.

There are evidence indicating that symbiotic interaction between some rhizobia and plants may involve plant lectins and bacterial LPS. The peanut root lectin PRA II binds to the LPS from several strains of *Bradyrhizobium* sp. [39], [40], and the PsNLEC-1 lectin-like protein from pea nodules binds to *Rhizobium leguminosarum* 3841 [41]. Furthermore, this lectin also binds to *Rhizobium leguminosarum* B551, *Sinorhizobium meliloti* EFB1, but not to *R. leguminosarum* B659, a LPS defective mutant [41].

The role of lectins in the interaction of gramineous plants with associative bacteria is poorly understood. Wheat germ agglutinin is present on surfaces of wheat seedlings and on root tips of adult wheat plants [42], and may therefore be a site for specific attachment of rhizobacteria contributing to bacterial adhesion to the root surface [43]. The previously uncharacterized maize GlcNAc-binding lectins identified in the present work have homologs in other grasses such as wheat, rice and sorghum, plants that are highly colonized by *H. seropedicae*. Indeed, lectins of several cereals, such as wheat, rye, barley and rice, are related and can be inhibited by GlcNAc and its oligomers [44]. The competition assays between *H. seropedicae* and *A. brasilense* showed that different bacterial species containing N-acetyl glucosamine in the capsule polysaccharides compete to colonize maize roots (and probably other grasses), and that the structure of such polysaccharides may contribute in determining the specificity of plant-bacteria interactions. It is likely that the interaction between LPS O-antigen and the MRLs is a key step for the establishment of the bacteria onto the root enhancing *H. seropedicae* attachment and conferring a competitive advantage during colonization of root surfaces, to gain access to inner tissues.

## Supporting Information

Figure S1 **Agglutination assay of** ***H. seropedicae*** **in the presence of WGA.** The wild type (A) and *waaL* (B) strains at OD_600_  = 1 were incubated with increasing concentrations of WGA (Sigma) or bovine serum albumin (BSA, control) during 60 minutes at 30°C in the absence or presence of 2% N-acetyl glucosamine (NAcGlc), 2% purified *H. seropedicae* wild type LPS or 2% glucose. Agglutination is indicated as the percentage of bacteria in suspension (as OD_600_) compared to the control ± standard deviation.
(DOC)

Figure S2 **Electrophoresis of maize root lectins purification fractions on a 12% SDS-PAGE.** Maize lectins were purified using an affinity chromatography on an N-acetyl glucosamine-agarose column as described in Experimental procedures. Lanes: MW - molecular weight markers (in kDa); 1: maize root crude extract; 2 – column flow-through; lane 3 – column wash fraction; 4 and 5 – protein fraction eluted with 0.5 M N-acetyl-D-glucosamine. MRL-1, MRL-2 and MRL-3 indicate maize root lectins. Proteins were stained with Coomassie blue.
(DOC)

Figure S3 **Structural models of MRL-1 and MRL-3. (A) MRL-1 or MRL-2 present a C-terminal digirent domain (brown) and a N-terminal jacalin domain (red).** The residues G188, T266, S309, R310, L311 and A313 (blue) constitute the putative N-acetyl glucosamine binding site. (B) MRL-3 presents two domains B chains (red). The protein domains and binding sites were identified with the program PFAM, the probable structures were generated by SwissModel.
(DOC)
