# Supplementary material for: Maize Root Lectins Mediate the Interaction with Herbaspirillum seropedicae via N-Acetyl Glucosamine Residues of Lipopolysaccharides
Source: PLoS One. 2013 Oct 9;8(10):e77001. doi: 10.1371/journal.pone.0077001 (PMC3793968; doi:10.1371/journal.pone.0077001)
Supplement: Figure S1 — Agglutination assay of H. seropedicae in the presence of WGA. The wild type (A) and waaL (B) strains at OD600 = 1 were incubated with increasing concentrations of WGA (Sigma) or bovine serum albumin (BSA, control) during 60 minutes at 30°C in the absence or presence of 2% N-acetyl glucosamine (NAcGlc), 2% purified H. seropedicae wild type LPS or 2% glucose. Agglutination is indicated as the percentage of bacteria in suspension (as OD600) compared to the control ± standard deviation. (DOC) [file pone.0077001.s001.doc]

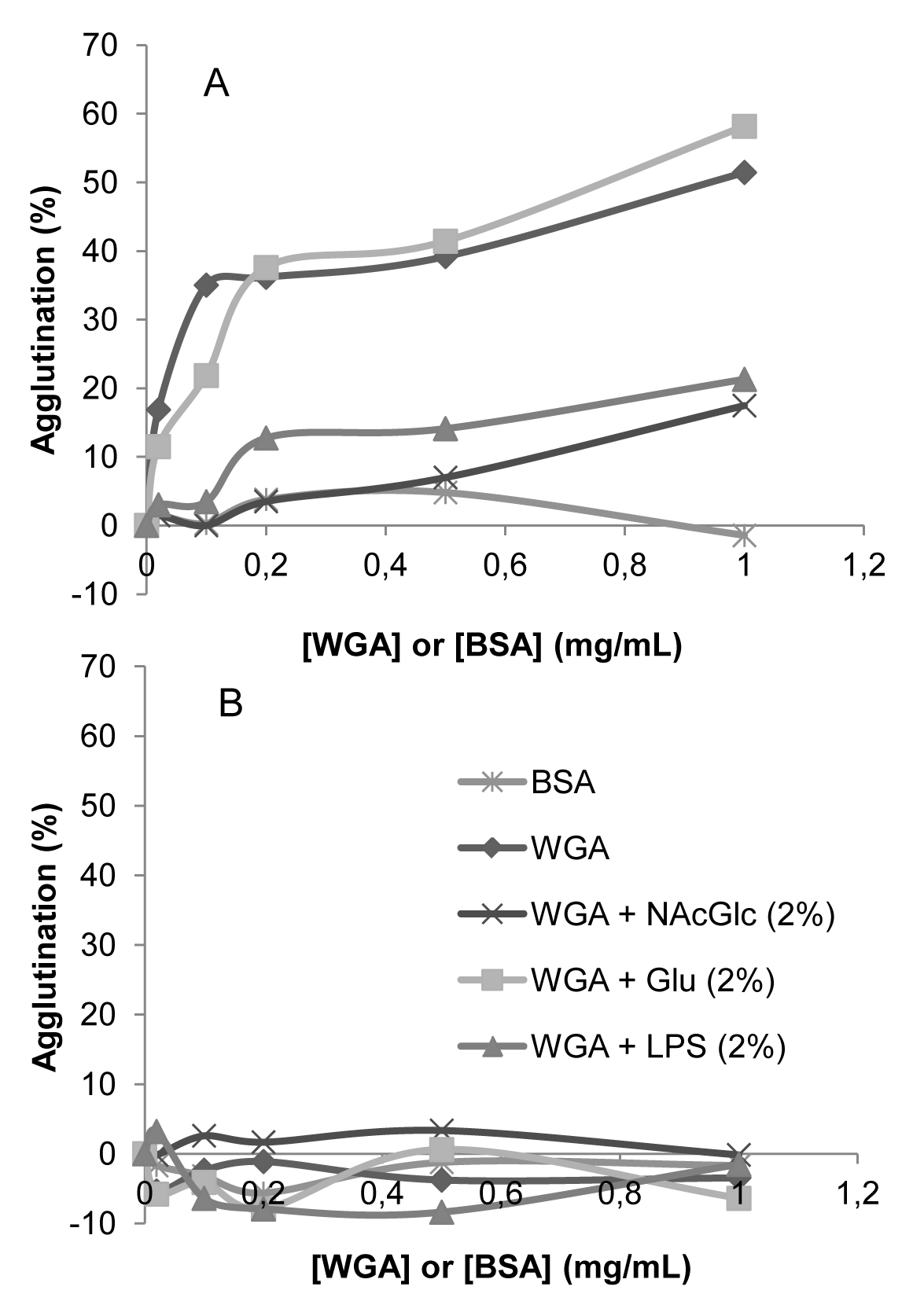


Figure S1 – Agglutination assay of *H. seropedicae* in the presence of WGA. The wild type (A) and *waaL* (B) strains at OD_600_ = 1 were incubated with increasing concentrations of WGA (Sigma) or bovine serum albumin (BSA, control) during 60 minutes at 30°C in the absence or presence of 2% N-acetyl glucosamine (NAcGlc), 2% purified *H. seropedicae* wild type LPS or 2% glucose. Agglutination is indicated as the percentage of bacteria in suspension (as OD_600_) compared to the control ± standard deviation.
