# Supplementary material for: Maize Root Lectins Mediate the Interaction with Herbaspirillum seropedicae via N-Acetyl Glucosamine Residues of Lipopolysaccharides
Source: PLoS One. 2013 Oct 9;8(10):e77001. doi: 10.1371/journal.pone.0077001 (PMC3793968; doi:10.1371/journal.pone.0077001)
Supplement: Figure S2 — Electrophoresis of maize root lectins purification fractions on a 12% SDS-PAGE. Maize lectins were purified using an affinity chromatography on an N-acetyl glucosamine-agarose column as described in Experimental procedures. Lanes: MW - molecular weight markers (in kDa); 1: maize root crude extract; 2 – column flow-through; lane 3 – column wash fraction; 4 and 5 – protein fraction eluted with 0.5 M N-acetyl-D-glucosamine. MRL-1, MRL-2 and MRL-3 indicate maize root lectins. Proteins were stained with Coomassie blue. (DOC) [file pone.0077001.s002.doc]

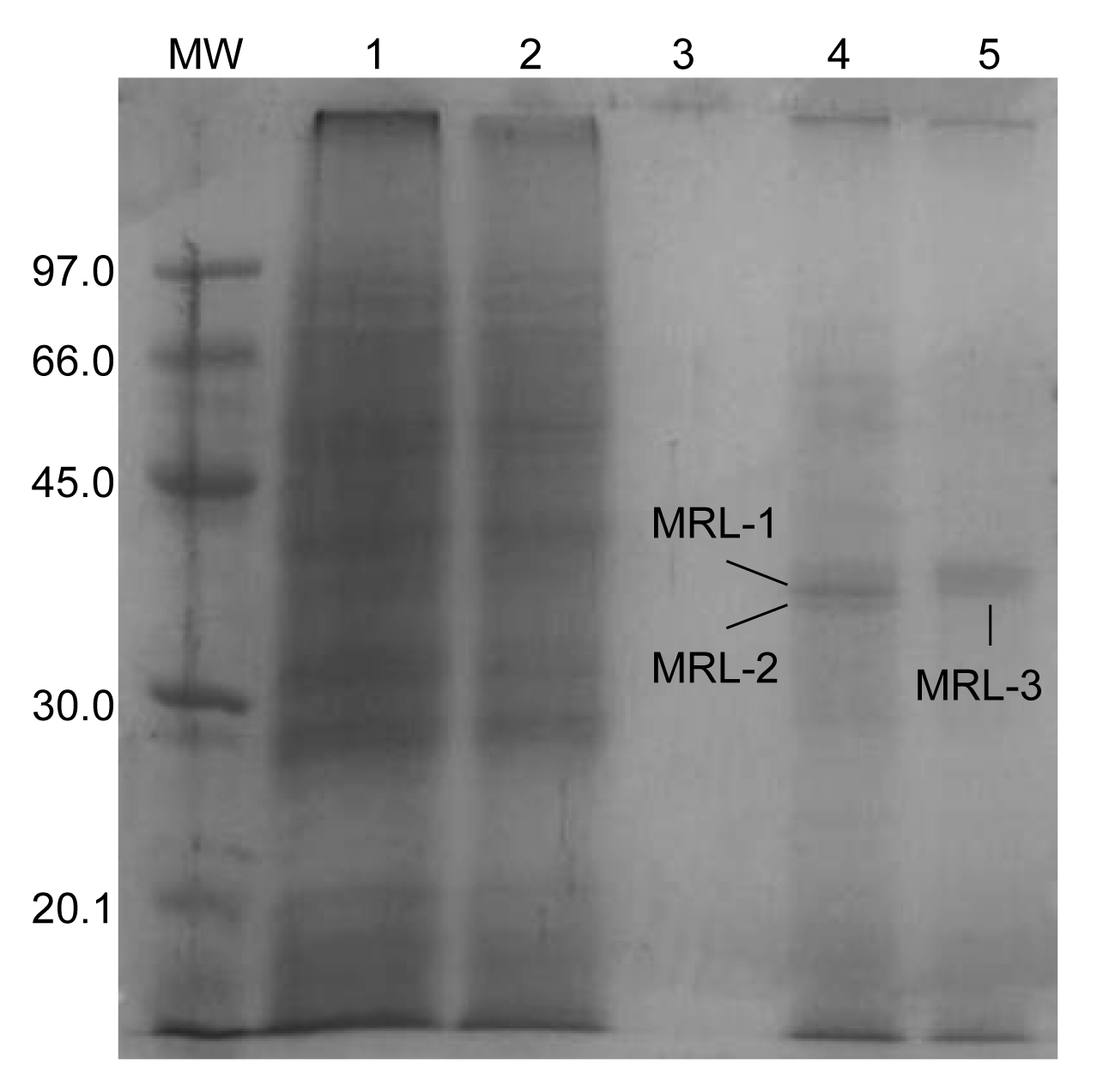


Figure S2 – Electrophoresis of maize root lectins purification fractions on a 12% SDS-PAGE. Maize lectins were purified using an affinity chromatography on an N-acetyl glucosamine-agarose column as described in Experimental procedures. Lanes: MW - molecular weight markers (in kDa); 1: maize root crude extract; 2 - column flow-through; lane 3 - column wash fraction; 4 and 5 - protein fraction eluted with 0.5 M N-acetyl-D-glucosamine. MRL-1, MRL-2 and MRL-3 indicate maize root lectins. Proteins were stained with Coomassie blue.
