# Supplementary material for: Maize Root Lectins Mediate the Interaction with Herbaspirillum seropedicae via N-Acetyl Glucosamine Residues of Lipopolysaccharides
Source: PLoS One. 2013 Oct 9;8(10):e77001. doi: 10.1371/journal.pone.0077001 (PMC3793968; doi:10.1371/journal.pone.0077001)
Supplement: Figure S3 — Structural models of MRL-1 and MRL-3. (A) MRL-1 or MRL-2 present a C-terminal digirent domain (brown) and a N-terminal jacalin domain (red). The residues G188, T266, S309, R310, L311 and A313 (blue) constitute the putative N-acetyl glucosamine binding site. (B) MRL-3 presents two domains B chains (red). The protein domains and binding sites were identified with the program PFAM, the probable structures were generated by SwissModel. (DOC) [file pone.0077001.s003.doc]

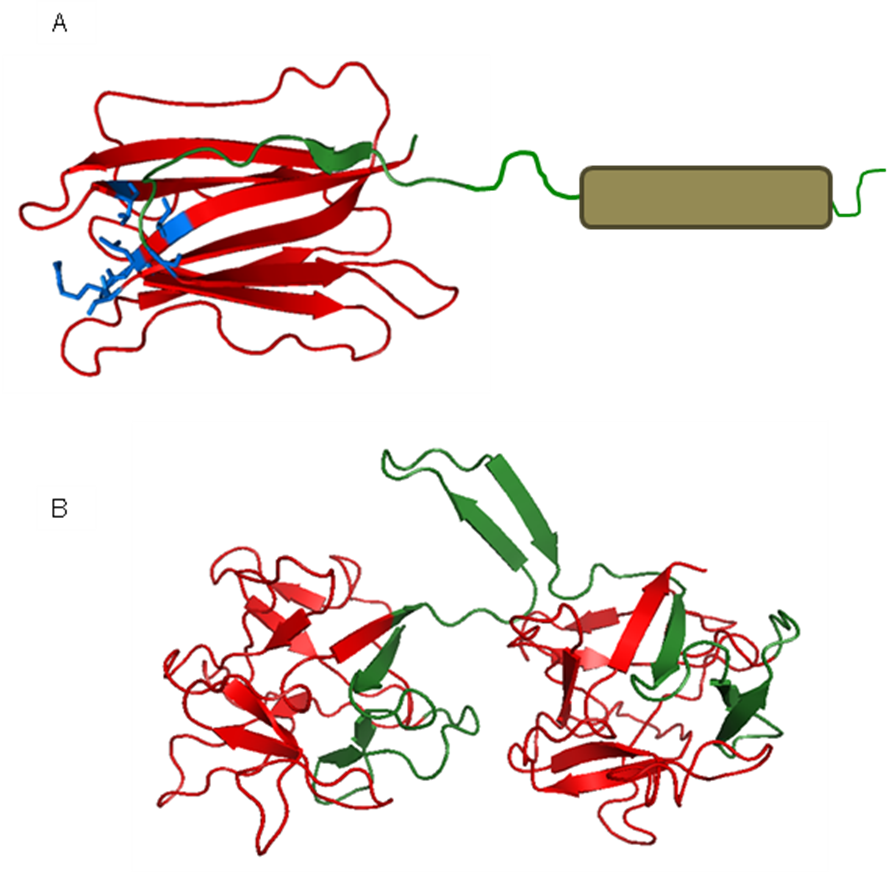


Figure S3 – Structural models of MRL-1 and MRL-3. (A) MRL-1 or MRL-2 present a C-terminal dirigent domain (brown) and a N-terminal jacalin domain (red). The residues G188, T266, S309, R310, L311 and A313 (blue) constitute the putative N-acetyl glucosamine binding site. (B) MRL-3 presents two domains B chains (red). The protein domains and binding sites were identified with the program PFAM, the probable structures were generated by SwissModel.
